# Supplementary material for: Bayesian inference is facilitated by modular neural networks with different time scales
Source: PLoS Comput Biol. 2024 Mar 13;20(3):e1011897. doi: 10.1371/journal.pcbi.1011897 (PMC10962854; doi:10.1371/journal.pcbi.1011897)
Supplement: Attachment — Submitted filename: reply_to_referee_report.pdf [file pcbi.1011897.s006.pdf]

Blue texts represent replies to reviewer comments.
Red texts indicate revised sentences corresponding to the comments.

Reviewer #1:

The authors present computational work showing that RNN can learn to represent prior and likelihood information in a close-to-optimal manner by obeying Bayes rule, and that this is accomplished by separated submodules that specialize to represent the prior with slow neurons and the likelihood with fast neurons. Overall, the paper is interesting and provide some novel results.

The introduction can be improved. It does not make justice to the interesting results that follow in the Results section. Also, in general the quality of English can be improved.

Thank you for your insightful comments. In response to your comments, we have thoroughly revised the introduction to ensure not only to improve language clarity but also to provide a clearer preview of the significance and results of the research.

I wonder what would be the optimal architecture in the limit where p_t (the transition probability) goes to zero. I would assume that in this limit no modular architecture is needed, as the prior is fix and does not change over time (and it can be stored in the weights of the network). Could the author perform simulations of this case, and compare to the limit of p_t going to zero, as in Fig. 3b?

Thank you for your important question regarding the optimal architecture as the transition probability p_t approaches zero. In the context of this study, signals are generated from different generators every 120 timesteps, so even in the case where $p_t = 0$, the situation effectively switches with a probability of 1/120, necessitating a modular structure. This is because, in this setting, p_t effectively takes on a finite value. However, if the generator were fixed throughout the learning process, a modular structure indeed would be unnecessary, as you pointed out in your comment.

More details describing the learning method is needed. It is unclear how learning works when the prior distribution is changed over time with probability p_t. Is the prior relearn at each step? Or one uses all the data to train the network? For how long episodes? If yes, the network will naturally adapt to the time scale of the changes, but more discussion is needed.

We have added a more detailed explanation of the learning method in the "Task" section of the paper. As mentioned in the text, even when the generator

switches, the signal is not reset. However, after a sequence of 120 timesteps, the signal is reset at that point. The learning then proceeds with the computation of the loss function. We have added the following sentences.

"When the generator in the model changes, the distribution is changed accordingly to which y_{true} conforms. As a result, the external input signal s fed into the RNN also changes. However, the signal is not reset immediately after the change in the generator. Only after a sequence of 120 timesteps, the signal is reset at that point. "

The authors claim in the discussion that in the brain different subnetworks are in charge of encoding priors and likelihoods. I think that the authors are right that generally speaking slow, high level variables are encoding in higher brain areas than fast, low level variables. However, there are some exceptions. For instance, in the work by Mochol et al, Current Biology, 2021, it is shown that prefrontal cortex encodes both priors and likelihoods in the same brain area. Therefore, I would rewrite the discussion and being more cautious about this.

Thank you for your insightful comments. Indeed, the findings from Mochol et al., Current Biology, 2021, differ from the results we cited the paper "Kording et al., Current Biology, 2012", where the prior and likelihood are encoded in different areas. In light of this, we have also referenced the paper by Mochol et al in the discussion and added further commentary to present a more nuanced view.

"However, caution is required as there are also experimental findings where the prior and likelihood are encoded in the same brain area¥cite{MOCHOL20211234}."

In Fig. 8, I would not use transparent colors to display the distributions.

We have revised Fig. 8 by removing the fill colors from the histograms and displaying only the outlines.

Line 3: animal brain -> animal brains

We have corrected as follows:

In the human and various animal brains, information processing involves inference based on inputs from the external world through the sensory systems, which obtain information from inputs under uncertainty due to noise.

Line 83: are the noise terms assumed to be independent

We have corrected as follows:

In Eq.¥ref{eq:regular_network_dynamics}, ${¥boldsymbol ¥xi}$ was used to account for independent noise in dynamics given by a random variable that follows a normal distribution with mean 0 and standard deviation 0.05.

Line 88-89: 'and' in cursive

We apologize for the oversight. We have made the correction.

Line 208: unclear why y depends explicitly on s. I thought that it would depend only on x_m explicitly.

We apologize for the confusion. You are indeed correct that y is determined by a linear transformation of x_m, and therefore depends on x_m at the same time step. What we meant to convey was that it depends on the values of s, x from the previous time step, and we have revised the text, as follows:

$y({¥bf x}_m(t-1;¥mu_g, ¥sigma_g),{¥bf x}_s(t-1;¥mu_g, ¥sigma_g), s(t-1), ¥sigma_l)$

Thank you again for your constructive comments. We believe that the revised manuscript meets your comments, and the manuscript is now acceptable for publication in PLoS Computational Biology.

Reviewer #2:

When performing Bayesian inference, the "prior" often changes abruptly, and for inference to be accurate these changes must be tracked. Here the authors investigate a modular network with both slow and fast timescales. They find an optimal timescale in the slow network. And, they also show that the timescale, and modular structure, can be learned, something that is very important.

Overall, this is an interesting paper that, I think, should be published. However, I got pretty lost in places, and the authors should clarify things before it's released to the public. The following comments should clarify my confusion (and also point out other issues, which were more minor).

Larger font on figures would be greatly appreciated -- some were *very* hard to read (because I'm old ;)).

Thank you for your positive evaluation. We have revised the manuscript to clarify the explanation overall as shown in red. Specific replies are as follows. We have increased the font size in the figures. Thank you for bringing this to our attention.

l 80, typo, I think: "= 0" --> "= z".

We have corrected the corresponding sentence as follows:

We adopted the activation function ReLU(${¥rm ReLU}(z)=0$ for $z ¥leq 0$ and ${¥rm ReLU}(z)=z$ for $z > 0$).

Fig. 3: the y-axes should start at zero, to make it easy to see how big the effect is.

We have corrected the figures so that y-axes start at zero.

Fig. 3 (especially blue points in panel b): why not do more runs to decrease the error bars?

We have conducted additional runs to increase the sample size and replaced the figure accordingly. Thank you for the suggestion.

l 168: Fig. 2b should be Fig. 3b, I think.

We apologize for the error. We have corrected it.

l 195-232: Here I got very lost. You talk about setting mu_g and sigma_g for each module. But you can't do that, right? Instead, all you can do is determine how the input is generated. So the analysis doesn't make sense to me.

We apologize for the confusion. You are indeed correct that in the learning setting, it is not possible to fix μ_g, σ_g for each module. However, for the sake of analysis, it is possible to set the states within the RNN and simulate them as a numerical procedure. We have added a more detailed explanation to clarify this point.

This change is made by saving the value of internal state ${¥bf x}{m,s}$ obtained by applying the input s created from the generator of $(¥mu_g', ¥sigma_g')$, and by changing the value of ${¥bf x}{m,s}$ to that value during RNN inference.

l 264: I got somewhat confused here as well. It seems to me that mu_p and sigma_p are internal values that are not directly accessible. If so, how can you find a transformation from x to mu_p (l 272)?

Thank you for your comment. You are correct that μ_p and σ_p are internal values that cannot be directly accessed. However, for the purpose of analyzing the results, we fixed μ_g, σ_g and considered the internal state x obtained when inputting the signal, assuming that it carries μ_g, σ_g as an internal representation. From this assumption, we calculated the transformation matrix. Thank you for pointing this out. We have added the following text.

To obtain the transformation matrix $W_{¥mu_p}$, we created a data vector M_g that gives the series $¥mu_g^1, ¥mu_g^2,⋯$ and a data matrix X that gives the series of the internal states $¥bf x^1, ¥bf x^2,..$ which is obtained from the input with fixed $¥mu_g$ as follows:

¥begin{equation*} M_g = (¥mu_g^1, ¥mu_g^2, ...)^{¥mathsf T}, ¥ X = ({¥bf x}^1, {¥bf x}^2, ...) ¥end{equation*}

Then, we seeked for the matrix $W_{¥mu_p}$ such that $M_g ¥simeq W_{¥mu_p}X$. Using the Moore-Penrose pseudo inverse, we first obtain the best-fit matrix as $W_{¥mu_p}=M_g X^¥dag$¥cite{penrose_1955}. Let $¥mu_p$ be the result of the transformation by $W_{¥mu_p}$. As Fig. ¥ref{fig:decoding}(a) shows, $¥mu_g¥simeq ¥mu_p$ is valid.

That said, the result of this analysis -- that alpha_s=0.1 uses more past information than alpha_s=1 -- is not surprising. But it doesn't explain why alpha_s=0.1 is better for rapid switches. Maybe that explanation was where I got lost?

Thank you for your insightful comment. We indeed were initially surprised to see ¥alpha_s=0.1 is better for rapid switch. The behavior you noted can be explained by how the internal state of the RNN responds to rapid switching. When generators switch rapidly, i.e., every 2-time steps, the RNN interprets this as a signal created with large variation and distinguishes it from noise. Here the network needs to code the variance. As shown in Fig. 6, the network with ¥alpha_s=0.1, slow sub-module can code the variance ¥sigma_g. This allows for quicker adaptation by prioritizing the observed signal over prior information. For a more detailed explanation, we have added the following text to the paper:

The trajectories of the internal state ${¥bf x}_{sub}(t)$ are plotted by the first and second principal components in Fig.¥ref{fig:adjust} for the cases in which generators A and B switch every 2-time steps and every 30-time steps. Generators A and B both have $¥sigma_g=0.04$. In the case of switching every 30 time steps, they were located in the region taken by the internal state when $¥sigma_g$ was small. In the case of switching every 2-time steps, they were located in the region taken by the internal state when $¥sigma_g$ was large (See Fig.¥ref{fig:adjust}). This distinction was achieved, because in the latter case the

generators switched so rapidly that the RNN recognized the signal as that created by a generator with a large variance. This enabled to switch $y(t)$ quickly because the information of the observed signal s was prioritized over the prior information when calculating the output y.

Thank you again for your constructive comments. We believe that the revised manuscript meets your comments, and the manuscript is now acceptable for the publication in PLoS Computational Biology.

Reviewer #3:

In the paper *Bayesian inference is facilitated by modular neural networks with different time scales*, Kohei Ichikawa and Kunihiko Kaneko explore how recurrent neural networks capture short and long timescales effects in a dynamic Bayesian inference problem. In particular, they study how a modular RNN architecture with different timescales for their respective neural activity dynamics can better predict a noisy stimulus than an RNN with a simpler architecture. The paper quantitatively assesses numerous features of the modular architecture, and overall demonstrates that the modular RNN learns to represent long-time scale, prior information in the slow timescale submodule, whereas the fast timescale module learns to focus on the immediate problem of identifying the noisy stimulus.

Review and general recommendations

Overall I did like aspects of this paper, and I think the questions the authors raise about the role of timescales in inference are quite interesting. Moreover, the simulations they perform take some steps towards answering them. Nevertheless, the paper in its present form has fundamental issues:

(i) Overall the paper feels underdeveloped. Although particular explanations are written well enough, a lot of the text feels meandering, incomplete, and hard to follow. The figures also suffer from often conveying relatively little information, and with poor formatting and hard to read text. As such, this is a frustrating paper to review, because although I think some of the content is good, I feel like it's conveyed quite poorly. I feel the authors sent off this manuscript prematurely.

Thank you for your positive evaluation. We apologize for the lack of the text clarify and frustration you felt. We have taken your comments seriously and completely revised the manuscript to improve the clarity of the model and the

results. We hope that the restructured text and figures will convey our findings more effectively.

(ii) Similarly, the simulations presented in the paper are extremely limited both in breadth and quantity. As a neural network paper where the simulations are presumably not compute-intensive, I would like to see much broader testing of the hyper parameters of the network, from timescales, to number of neurons, to switching probabilities. Can the authors provide cases where the modularity of the network is *not* necessary? If the authors wish to support their claims with simulations rather than analysis, then the simulations must be to a much higher standard.

We acknowledge the concerns raised regarding the breadth and quantity of our simulations, and have taken the following steps to address them:

1. Neuron Timescale Variation: We conducted additional simulations with a variety of neuron timescales, denoted by $¥alpha_s$.
2. Number of Neurons Ratio between Main and Sub Modules: We studied the performance of our model by changing the number of neurons ratio between the main module and the sub-module.
3. Trainable $¥alpha$ with Varying Switching Probability: We also carried out the simulation in which $¥alpha$ values are trainable. We obtained the resultant distribution $¥alpha$ by varying the value of switching probability p_t, and how the $¥alpha$ distribution depends on p_t.

The results of these additional simulations have been discussed in the main text and corresponding supporting figures and information.

We also checked that when $¥mu_g$ and $¥sigma_g$ are constant, the modularity of the network is not necessary, and the difference in the performance with and without modularity was not detected.

From our extended investigations, several key insights were gained:

Efficient Bayesian inference was successfully achieved when the number fraction was not excessively biased. With regard to the variation of p_t, we noted that as p_t increased, the peak against ¥alpha moved to a smaller side.

We have added the above remark in the revised text.

Overall, based on the current amount of content, it feels like a tightly written version of this paper could fit into 5 pages. I apologize that such a comment does not provide the authors with much guidance, but in its present form the paper feels too underdeveloped to identify exactly where to fill it in. These issues

prevent me from recommending the paper for publication, and the authors will have to undertake major revisions to prepare it for publication.

Thank you for your comment. As mentioned above, and as will be also shown below, we have explained our model and results with much care and clarity, and have made major revisions throughout the manuscript. (Still reducing the length is difficult, as the reviewers ask for a more detailed explanation)

Local comments

"when performing Bayesian inference by the brain." (p. 1) redundant

You are right. We have removed the redundancy. Thank you for pointing that out.

In performing Bayesian inference by the brain, the prior distribution must be acquired and represented by sampling noisy external inputs.

"uncertainty" (p. 2) please cite Sokoloski in Neural Computation, 2017

Yes, we apologize that the citation was missing. We have added the reference to Sokoloski in Neural Computation, 2017. Thank you for bringing this to our attention.

In the human and various animal brains, information processing involves inference based on inputs from the external world through the sensory systems, which obtain information from inputs under uncertainty¥cite{8016502} due to noise.

"the" (p. 3) a

We have replaced "the" with "a" in line 55.

"the results to be discussed are 76 not altered as long as both the numbers are sufficient (say 100 vs. 50, 150 vs. 150 for 77 fast and slow neurons)" (p. 4) a bit more detail

Thank you for pointing out this. We have revised the sentence to provide a clearer explanation:

Although we have mainly shown the results with a specific ratio of 150 fast neurons to 50 slow neurons, we found that the key results remained unchanged; In fact, we will show later that unless the number ratio is excessively biased (e.g., 10:240 or 240:10). As long as there are enough neurons in each category to

capture the underlying dynamics, good Bayesian performance is achieved. The condition may further depend on the details in the network complexity or specific interactions forms between fast and slow neurons, though, which will need further studies.

In this revision, we have conducted the simulation by varying Ns:Nm as (10,240),(50,200),(100,150),(150,100),(200,50),(240,10). Except in the case of biased number fractions, i.e., 240:10 or 10:240, good performance in Bayesian inference was achieved with the separation of roles, where slow sub-modules represent the variance of inputs. In the case of 240:10, such separation was not achieved, whereas in the case of 10:240, the number of slow submodules is too low to achieve the Bayesian inference. The results are described in the text lines 94-98 and Supporting Information.

“xmandxs” (p. 4) typo

“ α mand α s” (p. 4) typo

We have corrected the typos.

“Then” (p. 5) this connecting word is confusing. Does this sentence follow from the previous?

We apologize for the confusion in the writing. We have revised the text to clarify the connection between the sentences. The new text reads:

As mentioned in the Introduction, we set this task, as the RNN can make predictions when it performs Bayesian inference. To perform it, the prior distribution needed for Bayesian inference must be estimated from the observed signal so that it is close to the generator distribution.

${¥bf u}(t)$ for Eq.¥ref{eq:regular_network_dynamics} (or ¥ref{eq:modular_network_main},¥ref{eq:modular_network_sub}) is given by the Probabilistic Population Code (PPC)¥cite{Ma2006}.

“the ease of firing” (p. 5) tuning curve width?

As you pointed out, we refer to the tuning curve width; here, we have revised the text to clarify this point as follows:

Also, $¥sigma_{¥rm PPC}^2$ is a constant that represents the width of tuning curve which was set as $¥sigma_{¥rm PPC}^2=1/2$ in this study.

“Therefore, training was performed to minimize the mean squared error (MSE) between the neural network output y(t) and the true value ytrue(t)” (p. 5) this is a unrealistic assumption and should be justified more.

We acknowledge that providing the true value $y_{true}(t)$ for training might seem artificial. However, such settings with provide the true value have been utilized in previous studies, such as in the work by Kording et al. in Current Biology, 2012. For the purposes of this study, particularly to investigate the necessity of a modular structure and time-scale difference, it will be useful to adopt the simple and previously established settings. Then we can compare the present case with the standard homogeneous network case, even though it cannot fully replicate the real-world scenarios. We have added this remark.

“Hyperparameters” (p. 6) the amount of detail here is excessive. Focus on the key parameters

Thank you for your feedback. While we agree that the details may seem excessive, they were included to ensure the reproducibility of the numerical experiments. Instead, we categorized the parameters in the table as key and other parameters, and denote the former as bold.

“pt” (p. 6) Why isn't this variable on the other side?

We apologize for the error. We have put it on the left side of the table:

Switching probability of the generator distribution p_t

“Fig 4.” (p. 8) This figure only provides a weak demonstration of the point

Yes, Fig.4 is put to illustrate the point, rather than offering a strong demonstration. A quantitative evaluation of this point is given in Fig. 3(b) where the degree of adapting to rapid environmental changes is presented.

“The network with αs = 1 could not follow rapid input changes, whereas that with αs = 0.1 could estimate the input prior effectively.” (p. 8) it would be nice to have some kind of explanation of how the alpha s timescale compared with the timescale of the switching. Is there an optimal alpha s that depends on switch speed?

In response, we conducted experiments by varying p_t values at 0.01 and 0.03 while also adjusting the $¥alpha_s$ values within the range of 0.06 to 0.16 (specifically, 0.06, 0.08, 0.1, 0.12, 0.14, and 0.16). From these experiments, we did not observe any significant accuracy differences across varying $¥alpha_s$ values. The accuracy is not pronounced and changes little when

$¥alpha_s$ lies between 0.05 and 0.15, indicating that within this range, the performance of our network remains relatively stable regardless of the precise $¥alpha_s$ value. For $¥alpha_s ¥lessim 0.01$ or $¥gtrsim 0.2$, the error is increased as shown in Fig.3.

We have added the above result in the revised text and Supporting Information.

"Fig 6" (p. 10) Labels and text are tool small in figures. Normalize text sizes across all figures.

We have corrected the font sizes in Figure 6 and standardized them across all figures. Thank you for bringing this to our attention.

"SI for the case" (p. 11) supplementary information? Please improve reference

We apologize for the insufficient information. We have corrected the text to explicitly refer to "supporting information." Thank you for pointing this out.

When the generator changed rapidly, the variance of the prior was larger than the variance of the generator, as shown in the Supporting Information.

Information for the case with $¥alpha_s=0.1$.

"Effects of different time scales" (p. 11) This section seems far harder to read than it should be, given the simplicity of the point the authors are trying to make

We apologize for the confusion. In this section, we have investigated how past input information is embedded into the generated prior. For it we have assumed that the current mean of the generator estimated by the RNN can be written as a superposition of past signals $s(t)$, and calculated the coefficients to compare models with different α_s values. We have thoroughly revised the section for clarity, and we hope that it now conveys the intended point more effectively.

"To examine the impact of α s differences on Bayesian inference accuracy in detail, we considered how each model with α s = 1 and α s = 0.1 represents prior as a function of the input signal." (p. 11) At some point I would like to see key model hyperparameters thoroughly grid searched

Thank you for your comment. In this study, the key hyperparameter is α_s, so we compared the behaviors of RNNs with different values of α_s. As we have examined, MSE remains to be low for 0.05 < ¥alpha_s < 0.2. For ¥alpha > 0.5, it is distinguishably larger. Here, we have compared the case ¥alpha_s=0.1 as a

representative for nearly optimal value with that of ¥alpha_s=1(without time-scale separation).

“ak defined by Eq.14 is plotted against t, for the model with $\alpha_s = 1$ and $\alpha_s = 0.1$ using 3000 data points.” (p. 12) Figure text is unhelpful. It should be possible to have some understanding of the figure based on the caption

We apologize for the lack of clarity in the figure caption. We have revised it as follows:

a_k defined by Eq.¥ref{eq:approximation_of_mu_p} is plotted against k, for the model with $¥alpha_s=1$ and $¥alpha_s=0.1$ using 3000 data points. Note that a_k corresponds to the coefficient when r that the RNN estimates the mean of the current generatr as a superposition of past signals $s(t)$, given by $¥mu_p ¥simeq ¥sum_k a_k s(t-k)$. Then, we showed that the model with $¥alpha_s=0.1$ used information of much earlier past steps, in order to estimate prior information than the model with $¥alpha_s=1$.

“Then,” (p. 12) You mean to say this is what this section is going to be about? This makes it sound like you've already done it

We apologize for the confusion. We have revised the sentence to clarify the intention of this section:

In this section, we will investigate whether such a modular structure would emerge by training a neural network to predict y_{true} from a homogeneous structure network.

“normal RNN” (p. 12) reference earlier equations?

We apologize for the oversight. We have clarified this by revising it as:

We again used the same neural network model as Eq.(1).

“In summary, a modular structure, shown in Fig.1(b), emerged through learning alone.” (p. 12) The way this section is set up, it feels like it trivializes the earlier results of the paper. You could (and maybe should) simply start the paper with this section, show that 0.1 is optimal, and then analyze the results throughout the rest of the paper. I spent a lot of time in this paper trying to guess why the authors chose particular settings for various hyperparameters.

It might be possible to start the present manuscript from the case with a variable connection in which α_s may be chosen, and then analyze the mechanism. As you pointed out, starting from the case with a variable connection and timescale,

and then exploring why α_s is achieved, by examining if it is optimal, and why it is so is one possible way of presentation. Still, this may be harder to be understood for some (or most) readers. The variable timescale/connection model is more complicated than the fixed one (indeed, the timescales and connectivity are distributed, which makes the analysis harder). Hence, we first show the modular structure with $\alpha_s \cong 0.1$ is optimal and analyze its mechanism. Then, we examine if such a state is reachable (note if such optimal structure is reachable by learning is a harder step, so we explain this later). We strive to clarify these points in our revised manuscript. Thank you for your suggestion. Here we have added the following remark.

Here the finding that the modular structure performs good Bayesian inference does not necessarily imply that such structure can be obtained just by learning.

In this section, we examine if such a modular structure is reachable just by learning.

"Future research should investigate how this optimal time scale depends on the time scale of environmental changes." (p. 13) I think this research needs to be in this study

As mentioned in our previous response, upon testing with varying values of p_t, we found that the optimal value of $¥alpha_s$ does not sensitively change based on these differences. We deleted the above sentence, and instead we have added Supporting Information, to show that MSE is low for 0.06 ¥lesssim alpha ¥lesssim 0.16. Provided that alpha_s is maintained within a certain range, the differences in alpha_s values are absorbed through the variations in the trained weights, resulting in no discernible difference in accuracy. This indicates that the specific value of $¥alpha_s$ may be somewhat robust to environmental timescale changes, within the range we explored.

Thank you again for your constructive comments. We believe that the revised manuscript meets your comments, and the manuscript is now acceptable for publication in PLoS Computational Biology.
