# Supplementary material for: Bayesian inference is facilitated by modular neural networks with different time scales
Source: PLoS Comput Biol. 2024 Mar 13;20(3):e1011897. doi: 10.1371/journal.pcbi.1011897 (PMC10962854; doi:10.1371/journal.pcbi.1011897)
Supplement: Attachment — Submitted filename: reply_to_referee_report.pdf [file pcbi.1011897.s007.pdf]

Reviewer #2:

Thank you for your careful review and positive evaluation.

1. Typo in Eq. 2: should be N_m < i, not N_m ¥le i.
Thank you for pointing this out. We have corrected it.

2. How can you run with alpha=1? In that regime doesn't x diffuse off to infinity?

Thank you for your inquiry. In the case of alpha=1, the system simply turns to be a difference equation without "inertia". Even in this case, as the absolute values of eigenvalues of W are less than 1, x does not diffuse to infinity (as long as the noise is not too large) . In fact, according to the simulation, the divergence was not observed. Here we have included the definition range of alpha in the text for clarification.

Lines 88-90: where ¥textcolor{blue}{${¥bf I}$ represents ${¥bf I} = (1,1,...,1)^¥mathsf{T}$} and ${¥boldsymbol ¥alpha} = (¥alpha_1, ¥alpha_2, ..., ¥alpha_N)^¥mathsf{T}$ represents a vector to introduce the ¥textcolor{red}{rate of change, i.e. the inverse of} time scale of the neurons(¥textcolor{blue}{$0¥leq ¥alpha_i ¥leq 1$}). (Within this range of $¥alpha$, the present model works).

3. How often is u sampled? Every time step? Or only when things change?

It is sampled every time step. We have mentioned it in the revision as follows.

Lines 146-147: Accordingly, ${¥bf u}$ was sampled from the following Poisson distribution every time step

4. p_t is introduced on line 221, but I don't think you ever told us what it is.

Thank you for your insightful observation regarding the introduction of p_t. In response to your comment, we have revised the manuscript to clarify this aspect. Specifically, p_t was initially introduced at the beginning of the 'Task' chapter. To enhance the clarity and understanding, we have added an explicit explanation of p_t as follows.

Lines 127-129: Here it is important to note that the generator is not static, and is changed over time with a probability p_t, where p_t represents the parameter indicating the likelihood of changes in the generator.

6. Lines 238-9, "When a(t) is close to 1, the model’s prior is closer to generator B, and when a(t) is close to -1, it is closer to prior A." Shouldn't it be 0 and 1, not 1 and -1?

Yes, it should be 0 and 1. We have corrected it. Thank you for pointing this error out.

Lines 234-235: When $a(t)$ is close to 1, the model's prior is closer to generator A, and when $a(t)$ is close to 0, it is closer to generator B.

7. Fig. 2 is a bit unsatisfying. What we would like to see is a plot of y versus s for different values of mu_g and sigma_g. Along with a summary statistic.

Thank you for your valuable feedback. As you suggested, we have prepared a plot of y versus s for different values of mu_g and sigma_g, specifically for (mu_g, sigma_g) = (-0.3, 0.3), (0.3, 0.3), and (-0.3, 0.6)., and added some remark in the caption.

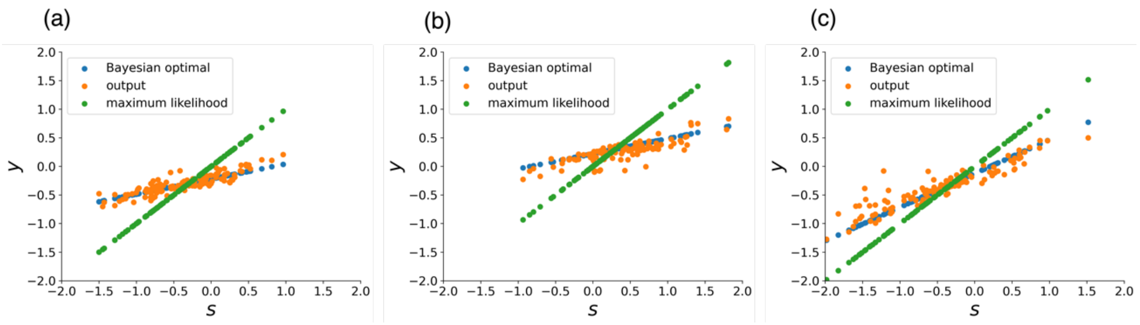

8. The mu_p explanation was confusing. As far as I can tell, you fit a matrix to mu_g = W_{mu_p} x, and then declared that mu_p = W_{mu_p} x? Is that correct? If so, you should say so; if not, I'm lost.

Yes, we have indeed defined mu_p as W_{mu_p}x. We have revised the text to make this definition clearer.

Lines 350-352: Here, there is the one-to-one relationship between the internal state $ {¥bf x} $ of the RNN and $ ¥mu_p $.

Given this background, we define $ ¥mu_p$ as $¥mu_p = W_{¥mu_p}{¥bf x} $.

9. Lines 362-4: "Here, note that the findings that the modular structure with slow/fast time scales works better for Bayesian inference do not necessarily imply that the structure can be achievable just by learning." I believe you're just saying that it's not clear that alpha can be learned. If so, you should say that. If not, I'm lost.

Thank you for your comment. In this chapter, the aim is to investigate whether learning can lead to the following properties simultaneously:

- The differentiation of alpha leading to fast and slow variables.
- The formation of a modular structure for neurons corresponding to these fast and slow variables.

Therefore, the focus is not merely on clarifying whether alpha can be learned, but also on whether the modular structure emerges corresponding to the differentiation of slow/fast variables

To enhance the clarity and understanding, we have revised as follows.

Lines 382-386: Here, it should be noted that our findings about the effectiveness of modular structures with slow/fast time scales in Bayesian inference do not necessarily imply such structure emerges through learning. In this section, we examine if slow/fast separation with corresponding modular structure is reachable just by learning.

Reviewer #3: This article is much improved, and the authors have addressed the bulk of my
concerns. Here are some additional comments to help further improve the manuscript.

Thank you for your careful review and positive evaluation.

p. 3, l. 44: "...corresponds to the higher layer in the brain." This statement needs to be rephrased and made more precise.

We have revised as follows.

Lines 44-46: Note that putamen, amygdala, insula, and orbitofrontal cortex in the brain have been found to represent prior in an experiment

p. 3, l. 59: "...the prior more appropriately than regular RNN." explain what you mean by this.

We have revised the text as follows.

Lines 57-58: We found that RNNs with a modular structure shape the prior more accurately than regular RNNs when the signal containing noise is inputted.

p. 3, Eq. 1: Make sure to introduce all variables in the equation, even if you can only fully define them later in the text.

We have revised the text as follows.

Lines 103-106: W_{in} and W represent the weight of synaptic connection. ${¥boldsymbol ¥xi}$ was used to account for independent noise in dynamics given by a random variable that follows a normal distribution with mean 0 and standard deviation 0.05.

p. 4, l. 153: You should state somewhere here exactly which parameters of the neural network you're optimizing.

We have revised the text as follows.

Lines 169-170: The training was performed by using the backpropagation method ¥cite{backpropagation_original, bptt}, to decrease L by optimizing the weight of synaptic connections W_*.

p. 10, l. 279: "...strongly reflects the difference in the prior distribution to the difference in output, respectively." This sentence is unclear.

We have revised the text as follows.

Lines 285-287: The magnitudes of V_s and V_m indicate the extent to which the sub-module and main module, respectively, influence the variation in the output, to respond upon changes in the signal's prior distribution.
